# Looking Ahead in Pulmonary Rehabilitation

**DOI:** 10.3389/fresc.2020.615545

**Published:** 2021-02-11

**Authors:** Enrico Clini, Stefania Costi

**Affiliations:** ^1^Department of Medical and Surgical Sciences, University of Modena and Reggio Emilia and University Hospital of Modena Policlinico, Modena, Italy; ^2^Department of Surgical, Medical and Dental Department of Morphological Sciences Related to Transplants Oncology and Regenerative Medicine, University of Modena and Reggio Emilia and Azienda Unità Sanitaria Locale, IRCCS, Reggio Emilia, Italy

**Keywords:** rehabilitation, COPD, chronic respiratory failure, programs, cardiac disease

## Introduction

Pulmonary rehabilitation (PR) is a comprehensive patient-tailored intervention particularly recommended for people suffering from several symptomatic respiratory diseases independent on their different functional stage and/or comorbid condition (1). Traditionally, PR consists of a structured multidisciplinary program including supervised exercise training, education, implementation of self-management strategies and other supports (e.g., nutrition, psychology, and mood disturbance). The program is formally delivered on a group-basis principle, either as inpatient, outpatient and even in the home setting (1).

Both experience- and evidence-based medicine inform consistently that PR is a cornerstone of treatment for patients with chronic obstructive pulmonary disease (COPD) (2, 3). More recently, PR models found to be effective in COPD were applied to other chronic respiratory conditions, such as symptomatic interstitial lung disease, bronchiectasis and pulmonary hypertension, and further extended to be early timely adopted at the very onset of worsening symptoms and disability, such as in the acute exacerbations and in the critical care area (1, 4).

This comprehensive model has proven to be of top level effectiveness in patients with COPD, in particular. Indeed, PR supports benefits in this population including better exercise capacity, reduced dyspnea, enhanced health-related quality of life and reduced admission to hospital (1). However, only indirect data seem to confirm a possible advantage in terms of survival, especially for those who suffered from severe exacerbations (5). Similarly, but inconsistently, the gains with PR programs delivered to patients with COPD could be also expected in respiratory condition other than COPD.

## What We have Learned to Date

Theoretically, all patients suffering from a chronic and symptomatic respiratory condition are potential candidates to PR based on the persistence and/or the worsening of their disability. However, several individual's multidimensional characteristics and profiles, and/or the level of disability and symptoms perception at baseline may influence the response to PR and its likely efficacy (6).

In the everyday clinical practice, important factors such as limited funding and reimbursement, lack of healthcare professional skills, poor awareness, and additional patient-related barriers influence worldwide the actual delivery of PR services to all the suitable patients (7).

Therefore, and from one side, PR is still grossly underutilized despite its important and recognized benefits with few individuals (less than 10% of eligible) ever undertaking a single program (8). Indeed, the American Thoracic Society (ATS) and European Respiratory Society (ERS) published a statement to address this implementation failure, warmly suggesting a call for new PR models in order to make this evidence-based treatment “*more accessible and acceptable both to patients and payers*” (9).

From the other side, since PR is a challenging intervention for patients, clinicians should improve their ability to screen for eligible patients at their best with the double purpose to identify those individuals who might benefit most from PR and to allocate the available resources properly.

Actual literature reports new data and new evidence with regard to the effectiveness of alternative PR models in patients with chronic respiratory diseases. As an example, tele-rehabilitation, home-based service at low cost, web-enabled or app-based activities have been proposed in recent years as means to provide different ways for gaining similar outcomes (10) with the scope to widen accessibility to PR programs.

Despite the potential of new PR models for implementation worldwide, the related programs have not been clarified as sufficiently comparable to the standard of care which is still a center-based program (11). In particular, the agreement of experts on both the indication, the delivery, the content, and the outcome set in these new PR models is still far to come (12).

To date, comprehensive, regular, and multidisciplinary assessment, including key outcomes for each specific patient, is necessary to provide a good basis for applying the most adequate interventions in PR and to monitor the patient's progression.

Exercise training, physiotherapy, physical activity and behavior change, occupational therapy, nutrition support, psychology, pharmacotherapy, education, and nursing, among the others, have an important role, so that patients benefit from a multifaceted care which should be ideally embedded in PR programs.

In other words, we do know that center-based PR as the standard of care properly works and fits with the right patients, but we do not know whether simplifying programs and contents in the so called new models in order to improve access might be really worth to the health care system or at least to the patient's rehabilitation tracks.

## What We Still Have To Do

The history of pulmonary rehabilitation is interesting. What appeared to be a field or uncertainty with obscure benefits is now supported by evidence graded as top level by experts. Notwithstanding, it is a general hope that the next years will provide even more novel and exciting pathways up to a wider role of PR as a futuristic version of the field.

Specific assessment for specific outcomes, long-lasting effects and maintenance of benefits in the individual patient, the most appropriate access to his/her very earlier onset of disability, overcoming potential barriers, increasing awareness of this science among people and professionals, embedding new technologies and e-health principles all represent actual frontiers to better explore for improvement right now (13).

Therefore, the emergence of new models of PR that may warrant the multidisciplinary characteristics in an ideal program presents new opportunities to expand the “choice” for patients, to better fit any individual need as for the ideal personalized approach of medicine, to widen the scope and access to this care option.

Several areas of improvement are tackling with this perspective, such as individual's factors that best suit with a specific model and program, the definitive role for hybrid or stepped programs as compared to the traditional one, the role for a standalone package in the more compromised or confined patients, the awareness and involvement with this top effective therapy for humans across the world (7, 9, 12).

## Conclusion

A new approach to the future of PR is needed, taking into account the emergence and the adoption of alternative models, the essential and desirable components, but still ensuring that the quality of outcomes is maintained.

This is an exciting time for PR which may bring new opportunities and allow to explore new areas for improvement. Overall, the future of PR will include more choices for patients and greater personalization of programs, while comprehensive patient's assessment should continue to be a landmark of all the programs and models.

## Author Contributions

EC conceived the paper structure and wrote and edited the paper. SC searched for information and wrote the paper. Both authors contributed to the article and approved the submitted version.

## Conflict of Interest

The authors declare that the research was conducted in the absence of any commercial or financial relationships that could be construed as a potential conflict of interest.
